# Heterozygous mutations in ZP1 and ZP3 cause formation disorder of ZP and female infertility in human

**DOI:** 10.1111/jcmm.15482

**Published:** 2020-06-22

**Authors:** Qiqi Cao, Chun Zhao, Xiaolan Zhang, Heng Zhang, Qianneng Lu, Congjing Wang, Yue Hu, Xiufeng Ling, Junqiang Zhang, Ran Huo

**Affiliations:** ^1^ State Key Laboratory of Reproductive Medicine Department of Histology and Embryology Suzhou Municipal Hospital Nanjing Medical University Nanjing China; ^2^ Department of Reproduction The Affiliated Obstetrics and Gynecology Hospital of Nanjing Medical University Nanjing Maternity and Child Health Care Hospital Nanjing China; ^3^ Department of Biological Sciences Purdue University West Lafayette IN USA; ^4^ Center for Global Health School of Public Health Nanjing Medical University Nanjing China

**Keywords:** ART, female infertility, mutation, zona pellucida

## Abstract

The human zona pellucida (ZP) is a highly organized glycoprotein matrix that encircles oocytes and plays an essential role in successful reproduction. Previous studies have reported that mutations in human ZP1, ZP2 and ZP3 influence their functions and result in a lack of ZP or in an abnormal oocytes and empty follicle syndrome, which leads to female infertility. Here, we performed whole‐exome sequencing in two probands with primary infertility whose oocytes lacked a ZP, and we identified a heterozygous mutation in *ZP1* (NM_207341:c.326G>A p.Arg109His), which is situated in the N‐terminus, and a heterozygous mutation in *ZP3* (NM_001110354:c.400G>A p.Ala134Thr), which is situated in the ZP domain. The effects of the mutations were investigated through structure prediction and in vitro studies in HeLa cells. The results, which were in line with the phenotype, suggested that these mutations might impede the function of cross‐linking and secretion of ZP proteins. Our study showed that the two mutations in *ZP1* and *ZP3* influenced the formation of the ZP, causing female infertility. Meanwhile, these data highlight the importance of the ZP1 N‐terminus in addition to the conserved domains for ZP1 function and ZP formation. Additionally, the patient with the ZP1 mutation delivered a baby following intracytoplasmic sperm injection (ICSI); thus, we suggest the targeted genetic diagnosis of *ZP* genes to choose appropriate fertilization methods and improve the success rate of assisted reproductive technology (ART) treatments.

## 1 INTRODUCTION

The zona pellucida (ZP) is an extracellular glycoprotein matrix that surrounds mammalian oocytes and embryos until the early stage of blastocyst development. ZP proteins have been detected in the oocyte and granulosa cells during the primordial follicle stage, and they increased with follicular development.^1^, ^2^ The ZP is involved in process critical for reproductive development, such as oogenesis, fertilization and preimplantation embryo development.^3^ During mammalian fertilization, the ZP regulates sperm‐oocyte binding, induces the acrosome reaction and engages in sperm‐egg interactions to avoid polyspermy.^4^, ^5^ Subsequently, the ZP protects the embryo during preimplantation development.

ZP proteins contain several conservative structural features, such as an N‐terminal signal sequence, a ZP domain (ZPD), a C‐terminal propeptide with a consensus furin cleavage site (CFCS) and a transmembrane domain (TMD). ZP1 and ZP4 also contain a trefoil domain.^6^, ^7^ The ZP of mice contains ZP1, ZP2 and ZP3.^8^ Mice lacking ZP3 and ZP2, which act as sperm receptors, produce oocytes without a ZP, and the female mice are completely sterile.^9^, ^10^ *mZp1^−/−^* females have eggs with abnormal ZPs, and the mice are subfertile due to early embryonic loss.^11^ The structures of murine and human ZPs are similar; both have long heterodimeric ZP2 and ZP3 filaments that are linked ZP1 homodimers. Another human protein, ZP4, was later discovered as another ZP protein.^12^ In clinical ART, morphological assessment of the zona pellucida is an important way to determine the quality of oocytes. ZP dysmorphology can include extracellular abnormalities such as a dark ZP, an irregularly shaped ZP or the absence of a ZP. Therefore, understanding the genetic mechanism behind abnormal zona pellucida formation and maintenance is significant for the success rate in IVF. Recent studies have shown that several mutations in *ZP1*, *ZP2* and *ZP3* cause abnormal zona pellucida formation.^13^, ^14^, ^15^, ^16^, ^17^

In this study, we identified a mutation within *ZP3*, c.400G>A p.A134T in a sterile female patient, and the mutation was further confirmed in her family characterized by a dominant inheritance pattern; further, we investigated a novel heritable mutation in *ZP1* (NM_207341:c.326G>A) that was found in a primary infertile female patient from whom 5 degenerated oocytes and three mature oocytes with no ZP were retrieved in an ICSI attempt. Surprisingly, the eggs of patient with *ZP1* mutation fertilized successfully and developed to the blastocyst stage in vitro in the absence of ZP to ensure its integrity. We further investigated the effect of these missense mutations by structure prediction and in vitro studies.

## 2 MATERIALS AND METHODS

### 2.1 Human subjects and ethics approval

Infertility patients were recruited from the Reproductive Medicine Center of the Affiliated Obstetrics and Gynecology Hospital of Nanjing Medical University. All blood samples from donors were obtained with informed consent. This study was approved by the Ethics Committee of the Nanjing Medical University (2018/651) updated in October 2018.

### 2.2 Genomic DNA extraction

Genomic DNA was extracted from peripheral blood with a RelaxGene Blood DNA System (Tiangen, DP319), and a NanoDrop 2000 spectrophotometer (Thermo Scientific) was used to determine the DNA concentration and quality.

### 2.3 Genetic analysis

Whole‐exome sequencing was used to identify candidate variants. Whole‐exome capture was performed using the Agilent SureSelect Human All Exon V6 (Agilent), and sequencing was carried out on the Illumina NovaSeq 6000 platform (Illumina) by Microanaly Genetech Co., Ltd (Anhui). We selected candidate variants with the following criteria: (a) present in the patient and her father, (b) absent in her mother and (c) had not been reported previously or had under 1% frequency in public databases (such as the 1000 Genomes database, the genome Aggregation Database (gnomAD) and the Exome Aggregation Consortium (ExAC) Browser). Subsequently, the candidate variant was validated by Sanger sequencing, and PCR amplification was performed using 2× Rapid Taq Master Mix (Vazyme, P222). The PCR products were sequenced by GenScript.

### 2.4 Vector construction

Wild‐type human ZP1, mutant ZP1 (p.Arg109His), wild‐type human ZP2, wild‐type human ZP3 and mutant ZP3 (p.Ala134Thr) were constructed and then recombined with the eukaryotic expression vector pcDNA3.1. An HA‐tag, a FLAG‐tag and a MYC‐tag were fused at both N‐terminus and C‐terminus of ZP1, ZP2 and ZP3, respectively. The vectors were constructed by GenScript.

### 2.5 Cell culture and transfection

HeLa cells were maintained in Dulbecco's modified Eagle's medium (DMEM, Life Technologies/Gibco, #11995073) supplemented with 10% foetal bovine serum (FBS) (Life Technologies/Gibco, #10270106), 100 U/mL penicillin, and 100 mg/mL streptomycin (Beyotime Biotechnology, C0222), and the cells were kept at 37°C with 5% CO_2_. Cells were transiently transfected for 6 hours using Lipofectamine 2000 reagent (Invitrogen, #11668019); then, cells were washed twice with PBS and maintained in serum‐free medium for 48 hours before harvesting.

### 2.6 Western blots

Cell culture supernatants were collected and concentrated with Amicon Ultra0.5 centrifugal filter devices (Millipore, UFC5010BK) according to the manufacturer's protocol. Cell lysates and concentrated supernatants were prepared with RIPA cell lysis buffer (Beyotime Biotechnology, P0013C). Protein concentrations were determined with a BCA Protein Assay (Beyotime Biotechnology, P0012). Sixty micrograms of protein was separated by 10% sodium dodecyl sulphate‐polyacrylamide gel electrophoresis before being transferred to polyvinylidene fluoride membranes (Millipore, IPVH00010). Non‐specific binding sites were blocked for 2 hours at room temperature with 5% non‐fat milk in Tris‐buffered saline containing 0.05% Tween‐20. Membranes were incubated overnight at 4°C with a dilution of the following antibodies: GAPDH (Abclonal, AC002), vinculin (Proteintech, #66305‐1‐lg), MYC (Cell Signaling Technology, #2278), FLAG (SIGMA‐ALDRICH, F7425) and HA (MBL, #561). After incubation with an anti‐immunoglobin horseradish peroxidase‐linked antibody (Invitrogen, #31430 and #31460) for 1 hour, the immune complexes were detected by enhanced chemiluminescence (FDBIO, FD8020). For densitometric analyses, protein bands on the blots were measured by ImageJ software.

### 2.7 Coimmunoprecipitation (Co‐IP) assays

Cells were harvested and lysed with lysis buffer on ice for 30 minutes. Then, the cells were centrifuged at 138000 *g* for 30 minutes at 4°C. Protein lysates were incubated with 80 µL of Protein A/G Magnetic Beads (Bimake, B23202) after preclearing the beads. Then, 8 µg of anti‐HA antibody (MBL, M180‐3) or 8 µg anti‐MYC antibody (Abcam, ab32) was added, and mix was subjected to gentle rotation overnight at 4°C. Western blots were performed after the beads were washed six times with lysis buffer.

### 2.8 ELISA

To detect the levels of secreted ZP1 and ZP3, ELISAs were used according to the manufacturer's instructions. We tested the ZP1 or ZP3 levels using 20 samples of medium from non‐transfected cells as negative samples. After the ZP1/ZP3 levels were calculated, the cut‐off value was calculated as the mean value of the negative samples plus 2 × SD. The specificities of the ELISA kits were determined as true negative rate. The cut‐off values of ZP1 and ZP3 in this study were 37.07 and 30.09 pg/mL. Cells were plated at 1 × 10^6^ per 100 cm^2^ dish, and cell culture medium was collected and centrifuged. ELISA reagents from Nanjing SenBeiJia Biotech Co., Ltd. (H2263 and H2304) were used to detect secreted ZP1 and ZP3 in supernatants. All assays were performed in duplicate and were repeated three times.

### 2.9 Homology modelling and structure prediction

Human and chicken ZP1 proteins share high sequence similarities; thus, the crystal structures of ZP1 (PDB: 6GF6) were employed as the template for homology modelling. The structure of the N‐terminal ZP‐N domain of human ZP1 was generated in the Swiss‐Model server using default parameters.^18^

### 2.10 Statistical analyses

Mean comparisons among groups were performed by 1‐way analysis of variance, and a multiple range least significant difference analysis was used for intergroup comparisons. All experiments in this study were conducted in triplicate. Derived values are presented as the means ± SEM. *P* values < .05 were considered statistically significant. All statistical analyses were performed with SPSS 16.0.

## 3 RESULTS

### 3.1 Clinical characterization

**FIGURE 1:**
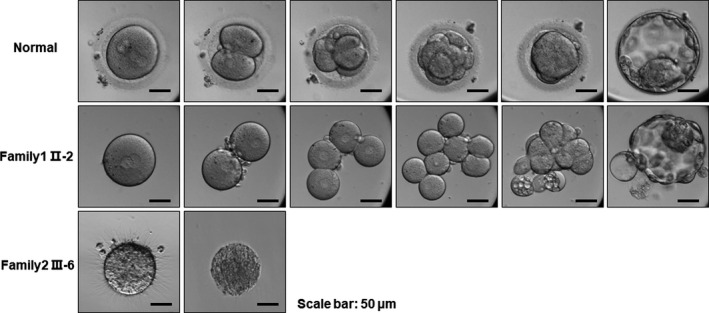
Clinical characterization. The oocytes retrieved from family 1 Ⅱ‐2 lacked a ZP, but they fertilized successfully and developed to the state of blastocyst in vitro during the ICSI treatment. The processes of early embryonic development were observed by time‐lapse microscopy. The oocytes retrieved from family 2 III‐6 lacked a ZP and degenerated the following day

The patient (family 1 Ⅱ‐2) had not conceived after 4 years of attempts without contraception, and she was diagnosed with primary infertility at the age of 29. She had regular menstrual cycles and normal sex hormone concentrations (Table 1). In the ICSI treatment attempt, a gonadotropin‐releasing hormone (GnRH) agonist protocol was performed. After an hCG trigger for 36 hours, oocytes were retrieved, and 8 cumulus‐oocyte complexes (COCs) were retrieved. Five oocytes degenerated after the cumulus cells were removed, and the other three oocytes developed to maturity but lacked a ZP. Subsequently, two of the mature oocytes were successfully fertilized and developed to the blastocyst state (Figure 1); however, one egg showed degradation following ICSI.

**Table 1:** Clinical features of the patients

Patient	Age	Primary infertility history	BMI	Basal sexual hormone				AMH (ng/mL)	Cycle			
				FSH^a^ (IU/L)	LH^b^ (IU/L)	E^c^ (pg/mL)	Testo (ng/dL)		No. of follicles on trigger day	No. of oocytes retrieved	No. of ZP‐free oocytes	No. of degenerated oocytes
Family 1 Ⅱ‐2	29	4	19.6	9.06	10.14	50	0.03	3.59	11	8	3	5
Family 2 Ⅲ‐6	28	5	18.4	7.13	3.08	61	0.66	2.02	8	0	0	0
									1	0	0	0
									9	4	2	2

The proband of family 2 (family 2 III‐6) had been diagnosed with infertility 5 years earlier; she had regular menstrual cycles and normal sex hormone concentrations, and she had undergone three cycles of IVF. No oocytes were obtained in the first two cycles, and 4 oocytes without a ZP were obtained from the 9 cumulus‐oocyte complexes in the third cycle, of which 2 degenerated shortly after retrieval and the other 2 oocytes died the following day (Table 1; Figure 1).

### 3.2 Heterozygous mutations in ZP1 and ZP3

**FIGURE 2:**
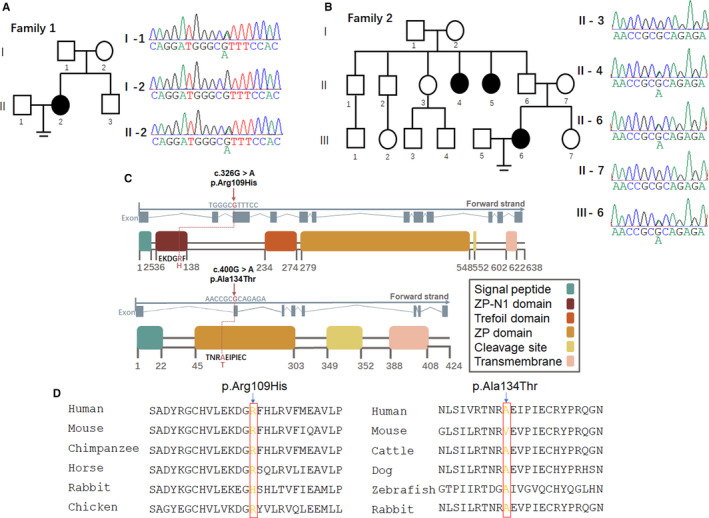
Heterozygous mutations in ZP genes. A, B, Left panel, pedigree of the patients. Male family members are represented by squares, and female family members are represented by circles; solid indicates affected members, and equal signs denote infertility. Right panel, Sanger sequencing results of the heterozygous missense mutation site in the patient (family 1 II‐2), her father (family 1 I‐1) and her mother (family 1 I‐2), and in the patient (family 2 III‐6), her fertile aunt (family 2 II‐3), her infertile aunt (family 2 II‐4), her father (family 2 II‐6) and her mother (family 2 II‐7). C, Location of the mutation site in *ZP1* and *ZP3*. The positions of mutations and functional domains are indicated in the gene structures. The *ZP1* missense mutation c.326G>A is in exon 3 of *ZP1*, causing the 109th amino acid, which is an R amino acid, to be replaced by an H amino acid. The *ZP3* missense mutation c.400G>A is in exon 2 of *ZP3*, causing the 134th amino acid, which is an A amino acid, to be replaced by a T amino acid. D, Conservation of mutated sites in *ZP1* and *ZP3* between species

To determine whether paternally transmitted mutations were involved in infertility, we performed whole‐exome sequencing of individuals in the family. We identified two mutations responsible for the phenotypes of these two patients. The variant of family 1 Ⅱ‐2, a heterozygous missense mutation c.326G>A (p.Arg109His) of *ZP1* (NM_207341) (Figure 2A, Table 2), could influence protein function since it is located in exon 3. Sanger sequencing was then used to validate the mutation (Figure 2A), and the patient (family 1 Ⅱ‐2) and her father (family 1 Ⅰ‐1) carried the heterozygous *ZP1* mutation, indicating a dominant pattern of inheritance, which may be involved in the ZP‐defective phenotype. The mutation was located in the N‐terminus of ZP1 before the trefoil domain (Figure 2C). The mutation in family 2 Ⅲ‐6, c.400G>A (p.Ala134Thr) in exon 2 of *ZP3*, had been identified in patients in a previous study.^16^ In addition to the previous study, we identified the mutation in a family instead of in sporadic individual cases. Sanger sequencing validated that the patient's father (family 2 Ⅱ‐6) and infertile aunt (family 2 Ⅱ‐4) carried the heterozygous missense *ZP3* mutation, whereas her fertile aunt (family 2 Ⅱ‐3) did not, indicating a dominant pattern of inheritance (Figure 2B). The altered amino acid substitution was located in the conserved ZP domain of ZP3 (Figure 2C). Moreover, the positions where these two mutations in *ZP1* and *ZP3* occur are highly conserved in most species (Figure 2D).

**Table 2:** Overview of mutations identified in the patients

Patient	Mutation gene	Genomic position (bp)	cDNA change	Protein change	Mutation type	Genotype	Mutation Assessor^a^	RefSNP ID	1KG_EAS^b^	ExAC_EAS^b^
Family 1 Ⅱ‐2	ZP1	chr19:60637017	c.G326A	p.R109H	Missense Mutation	Heterozygous	Possibly_damaging	rs369565345	NA	NA
Family2 Ⅲ‐6	ZP3	chr7:76058919	c.G400A	p.A134T	Missense Mutation	Heterozygous	Damaging	rs1554625334	NA	NA

Abbreviation: NA, Not Available.

^a^ Mutation pathogenicity prediction website: http://mutationassessor.org/.

^b^ Allele frequency in East Asian population of 1000 Genomes (1KG) and ExAC Browser.

### 3.3 R109H mutation in the predicted structure

**FIGURE 3:**
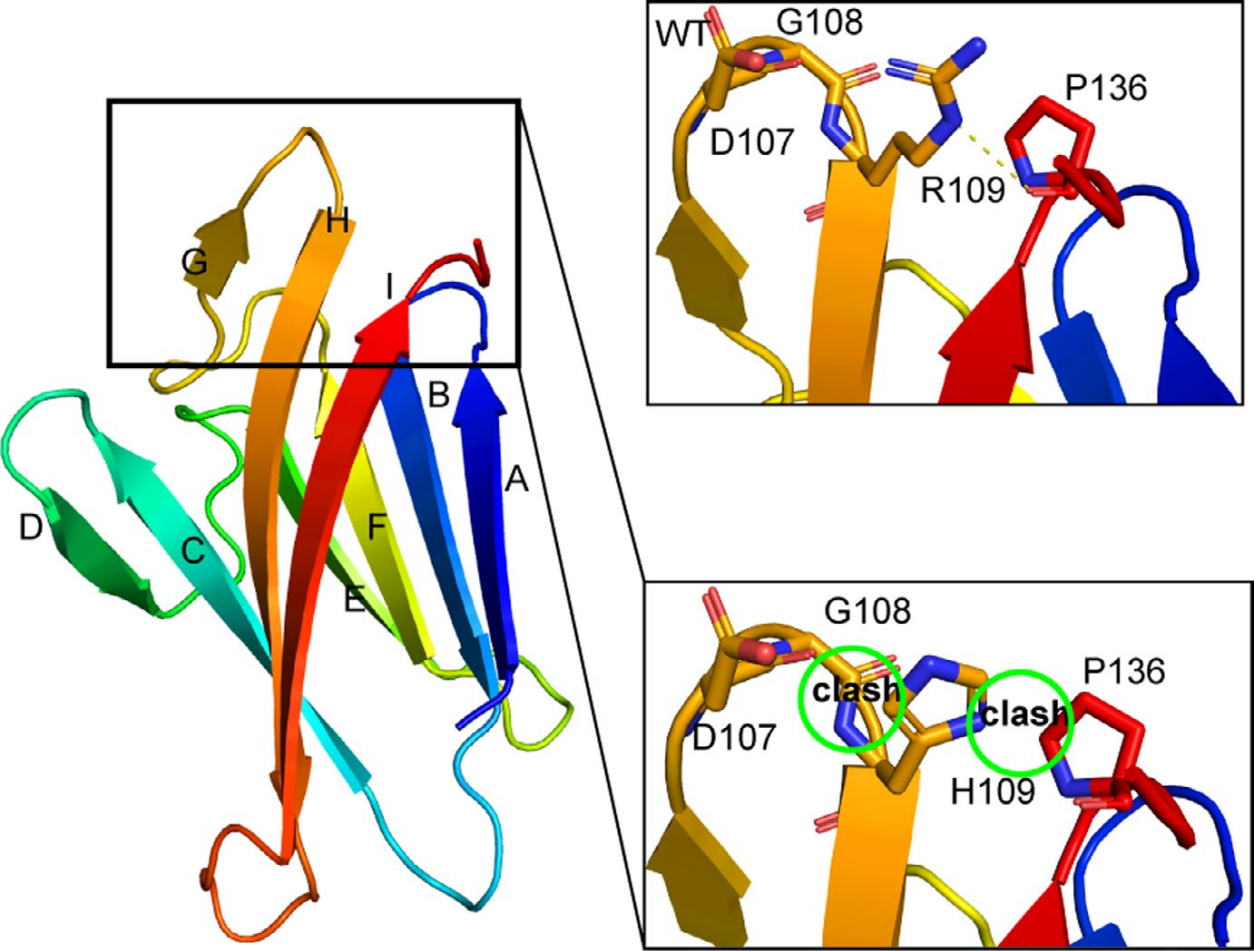
Structure prediction of the ZP1 mutation. Left panel: ribbon representation of the human ZP1‐N1 domain in rainbow coloration; right panel: close‐up view of residue 109 and its neighbouring residues

The A134T mutation caused altered structure of ZP3.^16^ In addition, to predict the molecular consequences of the novel mutation R109H in human *ZP1*, we performed homology modelling using the crystal structure of chicken ZP1. Not surprisingly, the overall structure of human ZP1‐N1 is similar to those of mouse ZP2‐N1 and ZP3‐N. Arg109 is located in strand H. The guanidino group of Arg109 would make hydrogen bonds with Pro136, thus stabilizing the protein structure. However, there would be a clash between the R109H substitution and neighbouring residues, including Asp107, Gly108 and Pro136, suggesting that the R109H mutation may affect protein stability or conformation (Figure 3).

### 3.4 Mutations in ZP1 and ZP3 affect interactions between ZP glycoproteins

**FIGURE 4:**
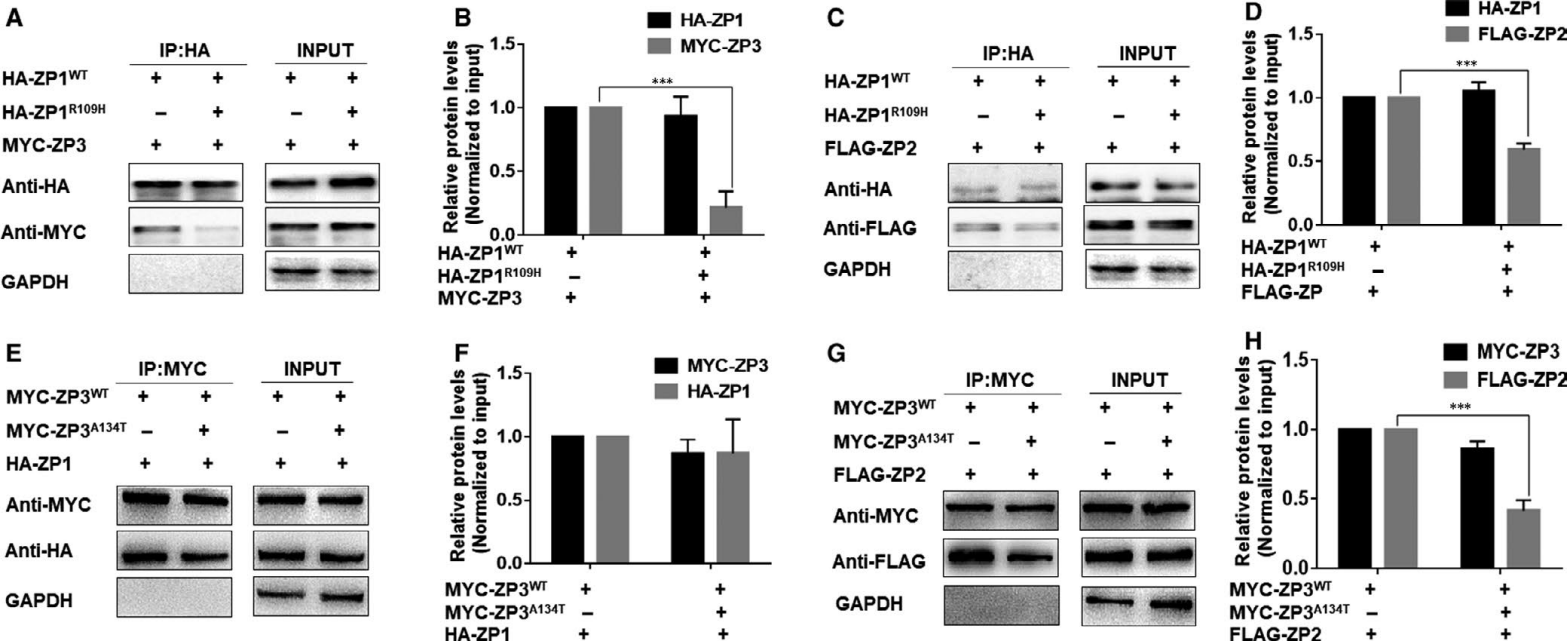
Interactions between ZP glycoproteins with mutated ZP1 and ZP3. A, B, Interaction of ZP1^WT^/ZP1^R109H^ with ZP3 or (C, D) ZP2 was evaluated by co‐IP with an anti‐HA antibody. E, F, Interaction of ZP3^WT^/ZP3^A134T^ with ZP1 or (G, H) ZP2 was evaluated by co‐IP with an anti‐HA antibody. Data are represented as the mean ± SEM; ***P < .001

To elucidate how these missense mutations led to ZP loss, wild‐type and mutant expression plasmids encoding human ZP1, ZP2, ZP3 proteins (HA‐tagged wild‐type human ZP1, HA‐tagged human mutant ZP1 (p.Arg109His), FLAG‐tagged human ZP2, MYC‐tagged human wild‐type ZP3, MYC‐tagged human mutant ZP3 (p.Ala134Thr) were co‐transfected into HeLa cells. We performed coimmunoprecipitation (Co‐IP) analysis to detect interactions between the mutant ZP1 and the other two ZP glycoproteins. When HA‐ZP1^R109H^ was co‐transfected with HA‐ZP1^WT^, FLAG‐ZP2 or MYC‐ZP3, imitating heterozygous mutations in the patient (family 1 II‐2), the expression of global ZP1 production was not affected when the mutated form is transfected, as shown in the input panel. Remarkably, the largely decreased interaction between ZP1 and ZP3 and slightly decreased interaction between ZP1 and ZP2 occurred when ZP1^R109H^ was transfected. As expected, ZP1^R109H^ diminished the interaction between ZP1 and ZP2 and ZP3 (Figure 4A–D).

When MYC‐ZP3^A134T^ was co‐transfected with HA‐ZP1, FLAG‐ZP2 or MYC‐ZP3 ^WT^, imitating heterozygous mutations in the patient (family 2 III‐6), the expression of global ZP3 production was not affected when the mutated form was transfected, as shown in the input panel. Remarkably, the largely decreased interaction between ZP3 and ZP2 occurred when ZP3^A134T^ was transfected (Figure 4E,F), which was in accordance with a previous study.^16^

### 3.5 ZP1 and ZP3 mutations disrupted secretion

**FIGURE 5:**
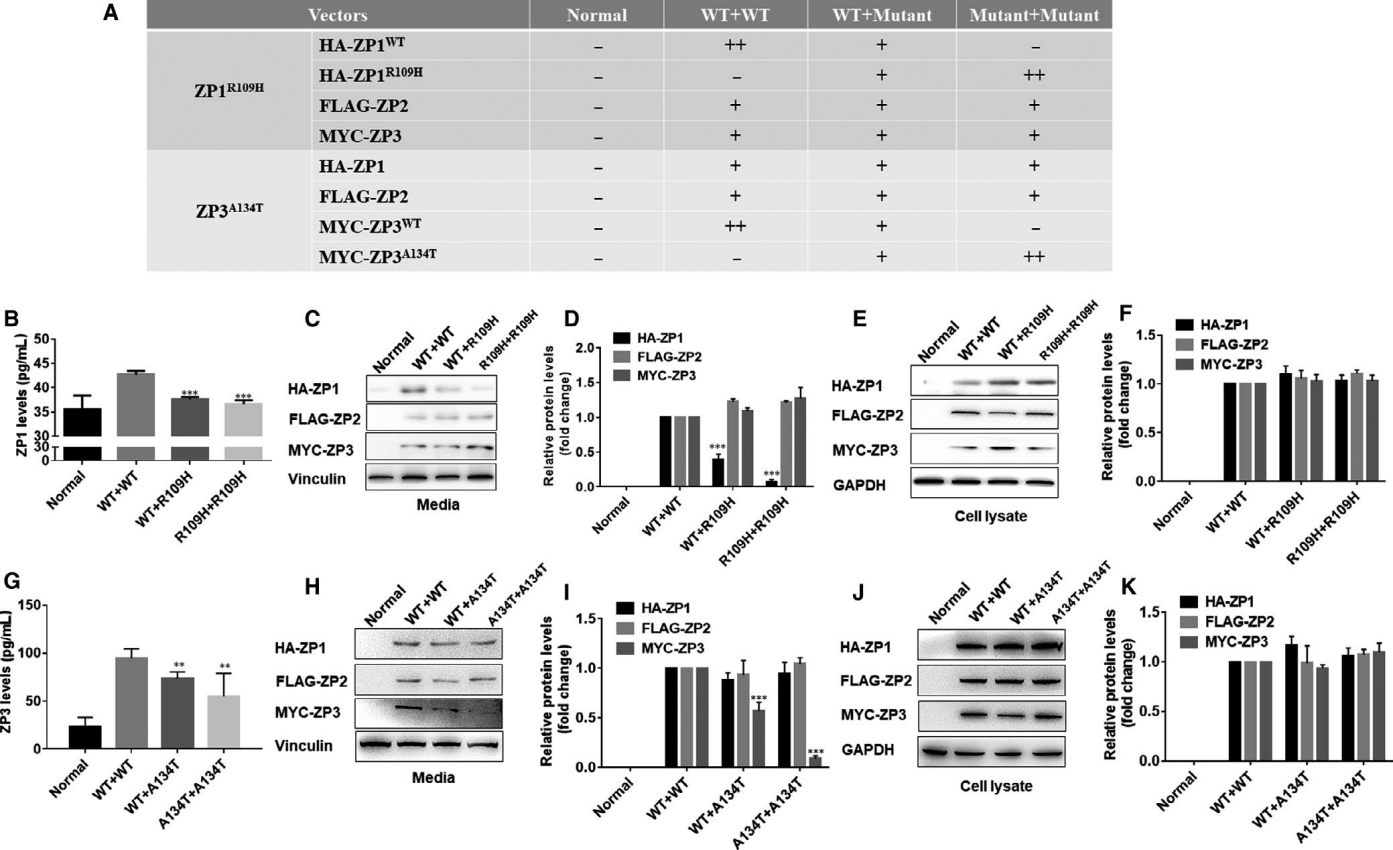
Effect of ZP1 and ZP3 mutations on ZP secretion. A, Transfection of vectors in different groups of cells. B, The ZP1 levels in the medium were measured by ELISA. C, D, ZP1, ZP2 and ZP3 protein levels in the medium were determined by Western blot. E, F, ZP1, ZP2 and ZP3 protein levels in cell lysates were determined by Western blot. G, The levels of ZP1 present in the medium were measured by ELISA. H, I, ZP1, ZP2 and ZP3 protein levels in the medium were determined. J, K ZP1, ZP2 and ZP3 protein levels in cell lysates were determined. Data are represented as the mean ± SEM; **P < .01 and***P < .001

We next asked whether mutations influenced the secretory function. To answer this question, we co‐transfected HA‐ZP1^WT^ or ZP1^R109H^ with FLAG‐ZP2 and MYC‐ZP3 to determine the influence of the ZP3 missense mutations on secretion. We co‐transfected MYC‐ZP3^WT^ or ZP3^A134T^ with FLAG‐ZP2 and HA‐ZP1 into HeLa cells and also had a group transfected with double mutant ZP1 or ZP3 to simulate homozygous mutations (Figure 5A).

Then, we collected the cell culture supernatants for assessment of secreted factors by a human ZP1 ELISA kit and a human ZP3 ELISA kit. We found that ZP1 and ZP3 levels were significantly decreased in the presence of mutant ZP1 and ZP3 compared to the levels of the control group (Figure 5B and G). Additionally, we used Western blotting to detect the expression level of ZP1, ZP2 and ZP3 protein in the cell lysate, and we detected their secretion levels in cell culture supernatants after co‐transfection. The results revealed that the secretion levels of ZP2 and ZP3 were comparable upon coexpression with either wild‐type ZP1 or mutant ZP1, whereas the levels of ZP1 in cell culture supernatants significantly decreased in the groups transfected with mutant ZP1 (Figure 5C and D), and ZP proteins were expressed normally in transfected cell lysates (Figure 5E and F). The bands indicated similar results when mutant ZP3 was co‐transfected (Figure 5H–K). Thus, these results showed that ZP1 with the p.Arg109His mutation and ZP3 with the p.Ala134Thr mutation interfered with the secretion of ZP proteins.

## 4 DISCUSSION

The ZP ensures the integrity of structure and participates in processes involved with fertilization, such as sperm‐oocyte recognition, binding and fusing.^19^ With the increase in ART application, oocyte quality and embryo development are being increasingly observed and investigated. The zona pellucida is clinically used as one of the indicators of oocyte quality. ZP dysmorphology, such as dark zona pellucida or the lack of a zona pellucida, has an incidence of 2%‐5% of all oocytes.^20^, ^21^ ZP dysmorphology has been reported to be associated with a significant reduction in pregnancy rates and implantation rates in IVF.^22^ However, the mechanism of ZP dysmorphology is rarely reported in the literature. In this study, we identified two infertile patients who had oocytes retrieved for ART treatments, and clinical observation revealed that the oocytes lacked a ZP, suggesting that the loss of the zona pellucida was the cause of their infertility.

As the ZP is quite important in fertilization, mutations in *ZP* genes may affect the zona pellucida and cause infertility. Recent studies have reported several cases of primary infertility caused by abnormalities in the zona pellucida.^13^, ^14^, ^15^, ^16^, ^17^, ^23^A mutation of *ZP1* (I390fs404X) causing the protein to be truncated was detected in an infertile patient whose oocytes lacked a zona pellucida.^14^ Another study identified a heterozygous mutation in *ZP2* (NM_003460.2:c.2092C>T) and another in *ZP3* (NM_001110354.1:c.1045_1046insT) in a patient diagnosed with primary infertility, and her retrieved oocytes had a thin or absent ZP.^15^ A recent study reported that two homozygous mutations in *ZP2* (c.1695‐2A>G, and c.1691_1694dup) led to a thin ZP and influenced sperm‐binding.^17^ Recently, several mutations in *ZP1* (three homozygous mutations: c.1708G>A, p.Val570Met; c.1228C>T, p.Arg410Trp; c.507del, p.His170Ilefs*52, and two compound heterozygous mutations: c.1430+1G>T, p.Cys478X and c.1775‐8T>C, p.Asp592Glyfs*29), *ZP2* (c.1115G>C, p.Cys372Ser) and in *ZP3* (c.763C>G, p.Arg255Gly) were characterized in patients with similar phenotypes.^13^ Our study identified a heritable heterozygous mutation in *ZP1* (c.326G>A p.Arg109His) from the patient (family 1 Ⅱ‐2) and her father (family 1 Ⅰ‐1), suggesting that this ZP1 mutation is associated with ZP loss and infertility in this patient. In addition, we recruited a family with female primary infertility and speculated that it was due to dominant inheritance. Then, we identified a previously reported *ZP3* mutation (c.400G>A p.Ala134Thr) that was associated with ZP‐free oocytes and degeneration.

Many studies have been carried out on the secretion and assembly of ZP glycoproteins using mouse models to date. The mouse ZP proteins are ZP1, ZP2 and ZP3. The human ZP proteins are ZP1, ZP2, ZP3 and ZP4. All four zona pellucida proteins present high homology and have common structural features such as signal peptide, ZP domain, CFCS, transmembrane‐like domain and short cytoplasmic tail. ZP1 and ZP4 also contain the trefoil domain. These domains have been extensively investigated; for example, the signal peptide and CFCS are associated with secretion. Additionally, the ZP domain is known to be responsible for regulating the combination of ZP2 and ZP3 with ZP filaments, and it has a functional activity and plays a role during fertilization.^24^ It is worth noting that the reported ZP mutations mentioned above are mostly located in the ZP domain and ZP‐C‐terminal; instead, in our study and another recent study the mutation of ZP1 lies in the ZP‐N1 domain of hZP1, and the ZP‐N1 domain lies in the N‐terminal of ZP1, which may harbour its cross‐linking function.^25^ According to Jovine's work, it was not the trefoil or ZP module region of human ZP1 that formed cross‐links; it was human ZP1‐N1. ZP1‐N1 was essential for the assembly of the human ZP and fertility. In line with this, our results indicated that ZP1 with the p.Arg109His mutation exhibited structural instability or conformational changes that affect cross‐linking and impede the formation of the ZP. Thus, it is suggested that mutations to ZP1‐N1 may also participate in the ZP formation, thus affecting the function of the zona pellucida and resulting in infertility.

In human ZP assembly, ZP1 cross‐links three other ZP proteins to form long filaments.^26^, ^27^ This finding was consistent with the phenotype of a mouse model in which *mZp2^−/−^* and *mZp3^−/−^* females produced oocytes completely lacking a ZP, and oocytes from *mZp1^−/−^* females were surrounded by a loosely organized and abnormal ZP.^11^, ^28^, ^29^ As reported, the *ZP3* mutation (c.400G>A p.Ala134Thr) could influence the link between ZP3 and ZP2, and the interruption of their connection could destroy ZP assembly, as confirmed by the results in our research.^16^ In the present study, we analysed the *ZP1* mutation through structural prediction. The substitution of amino acids caused by the mutation resulted in conversion of an arginine residue to a histidine. We found that the alteration is likely to be significant because it causes structural instability to affect the cross‐linking or binding function of proteins. Here, in vitro experiments in HeLa cells showed that mutant ZP1 reduced the interaction with ZP2 and ZP3. Taken together, we propose that this mutation influences the cross‐linking function of ZP1 and impedes the formation of ZP.

Zona pellucida is a glycoproteinaceous matrix that is synthesized in oocytes and secreted during follicular development.^30^ ZP proteins are known to be synthesized and secrete independently, and then ZP2‐ZP4 heterodimers are formed via cross‐linking by ZP1 homodimers.^24^, ^27^ It is thought that if the ZP proteins are not secreted appropriately, then the formation of the zona pellucida will be affected. It was reported that mutant ZP2 and ZP3 accumulated in the endomembrane system and could not bind to the cell membranes, resulting in a traffic barrier and the absence or thinness of the ZP.^15^ Hence, in our study, we used ELISA and Western blot experiments to detect the secreted levels of ZP1 and ZP3, both of which after introducing mutant forms of the proteins, and they also presented a mutation dosage‐dependent effect. The results indicate that the mutations are involved in the reduced secretion of ZP1 and ZP3, and leading to connection failure of the ZP filaments in vitro. The data suggest a potential that the mutations may be involved in the lacking ZP phenotype, which need to be further investigated in vivo.

Although the patient (family 1 Ⅱ‐2) had retrieved oocytes lacking a ZP, after undergoing intracytoplasmic sperm injection (ICSI) with a mature in vitro culture system, she successfully delivered a baby with an Apgar score of 10. This case suggests that ZP1 is an essential element of the zona matrix. Fertility is a complex process, and ZP1 plays important roles in fertilization, but the mechanism underlying this remains largely undiscovered. In the clinic, when encountering infertility patients who seek ART for help, we suggest that the *ZP* genes sequencing should be advised if a ZP anomaly is visible. If the sequencing result reveals a mutation in *ZP1*, the method of ICSI could be preferentially chosen instead of the traditional IVF method, which would greatly improve the probability of successful pregnancy via ART in infertility patients who have a ZP problem.

In conclusion, our study identified a *ZP1* mutant situated in the ZP1‐N1 segment upstream of the trefoil domain, ZP domain, cleavage site and so on. The mutation in this region impeded the cross‐linking and secretion function and may account for the absence of the ZP. It also suggested that human ZP1‐N1 also engages in the formation of ZP in addition to the domains of known functions such as the ZP domain. Moreover, the confirmation of a previously identified *ZP3* mutation emphasized its pathogenicity and phenotypic consistency. Our work increases the understanding of the pathogenesis of *ZP1* and *ZP3* gene mutations and recommends selecting a rational fertilization method of ART in combination with ZP target gene diagnosis for infertility patients in the clinic carrying *ZP1* mutations and ZP‐free oocytes.

## CONFLICT OF INTEREST

The authors confirm that there are no conflicts of interest.

## AUTHOR CONTRIBUTION

**Qiqi Cao:** Conceptualization (lead); Data curation (equal); Investigation (lead); Methodology (lead); Validation (equal); Writing‐original draft (equal); Writing‐review & editing (equal). **Chun Zhao:** Conceptualization (equal); Data curation (equal); Investigation (equal); Writing‐original draft (equal). **Xiaolan Zhang:** Conceptualization (equal); Formal analysis (equal); Resources (equal); Validation (equal); Visualization (equal); Writing‐original draft (equal). **Heng Zhang:** Software (equal); Visualization (equal). **Qianneng Lu:** Investigation (equal); Methodology (equal). **Congjing Wang:** Methodology (equal); Validation (equal); Writing‐review & editing (equal). **Yue Hu:** Formal analysis (equal); Investigation (equal). **Xiufeng Ling:** Project administration (equal); Resources (equal); Supervision (equal). **Junqiang Zhang:** Project administration (equal); Resources (equal); Supervision (equal). **Ran Huo:** Funding acquisition (lead); Project administration (lead); Supervision (lead); Writing‐original draft (equal); Writing‐review & editing (equal).

## Data Availability

The data used to support the findings of this study are available from the corresponding author upon request.
